# Role of G-protein-coupled Receptor-related Genes in Insecticide Resistance of the Mosquito, *Culex quinquefasciatus*

**DOI:** 10.1038/srep06474

**Published:** 2014-09-29

**Authors:** Ting Li, Lena Liu, Lee Zhang, Nannan Liu

**Affiliations:** 1 Department of Entomology and Plant Pathology, Auburn University, Auburn AL 36849, USA; 2 Department of Biology, Cornell University, Ithaca, NY 14853, USA

## Abstract

G-protein-coupled receptors regulate signal transduction pathways and play diverse and pivotal roles in the physiology of insects, however, the precise function of GPCRs in insecticide resistance remains unclear. Using quantitative RT-PCR and functional genomic methods, we, for the first time, explored the function of GPCRs and GPCR-related genes in insecticide resistance of mosquitoes, *Culex quinquefasciatus*. A comparison of the expression of 115 GPCR-related genes at a whole genome level between resistant and susceptible *Culex* mosquitoes identified one and three GPCR-related genes that were up-regulated in highly resistant *Culex* mosquito strains, HAmCq^G8^ and MAmCq^G6^, respectively. To characterize the function of these up-regulated GPCR-related genes in resistance, the up-regulated GPCR-related genes were knockdown in HAmCq^G8^ and MAmCq^G6^ using RNAi technique. Knockdown of these four GPCR-related genes not only decreased resistance of the mosquitoes to permethrin but also repressed the expression of four insecticide resistance-related P450 genes, suggesting the role of GPCR-related genes in resistance is involved in the regulation of resistance P450 gene expression. This results help in understanding of molecular regulation of resistance development in *Cx. quinquefasciatus.*

The development of insecticide resistance is a serious practical problem associated with the chemical control of disease-borne vector insects. One such insect is the mosquito, *Cx. quinquefasciatus*, which is a primary vector of the pathogens responsible for West Nile encephalitis, eastern equine encephalitis, Saint Louis encephalitis, and lymphatic filariasis and inhabits tropical and subtropical regions worldwide^1^,^2^,^3^. Pyrethroids are particularly suitable for controlling mosquito vectors and, consequently, for controlling against mosquito-borne diseases. However, the development of resistance to pyrethroids in mosquitoes has become an urgent and widespread problem for mosquito-borne disease control efforts worldwide^4^,^5^,^6^. Pyrethroid resistance has been found in a number of *Culex* mosquito species^7^,^8^, so a better understanding of the molecular basis of resistance mechanisms should provide new strategies for mosquito control. Cytochrome P450s play a pivotal role in insecticide detoxification in mosquitoes via an increased expression level in resistant mosquitoes^9^,^10^,^11^,^12^. Studies on P450 gene up-regulation or induction in response to xenobiotics have indicated that *cis-* or *trans*-acting regulators are involved in transcriptional regulation in the house fly^13^,^14^,^15^. Transcriptional element regulated xenobiotic induction of P450 genes has been functionally characterized in several other insect species. These includ EcRE/ARE/XRE-xan elements of *Cyp6b* promoters in tiger swallowtail^16^ and black swallowtail^17^ butterflies, the EcRE element of *Cyp6b* in black swallowtail butterflies^18^, the CuRE element of *Cyp9m10* in mosquitoes^19^, and the XRE-Fla element of *Cyp321a1* in corn earworms^20^, and the CncC element of *Cyp6a* has been shown to be involved in phenobarbital (PB) induction in fruit fly, *Drosophila* *melanogaster*^21^. Bhaskara et al.^22^ reported that the caffeine-inducible promotion of *Cyp6a* genes was probably regulated through the cAMP pathway in *D. melanogaster*. However, the up-stream regulatory pathway of P450 up-regulation or induction in insects remains largely unknown.

G-protein-coupled receptors (GPCRs) are integral membrane proteins with seven α-helical hydrophobic domains that carry out specific physiological functions^23^. They are divided into several subfamilies based on sequence similarity^24^. The function or dysfunction of GPCRs may cause various changes in cellular response, so GPCRs are major drug targets for a wide range of diseases and play a critical role in the development of new medical treatments for humans^25^,^26^. In insects, GPCRs have been shown to regulate physiological pathways^27^ and affect insect behavior^28^, reproduction^29^, development^30^,^31^, and metabolism^32^. Moreover, recent studies have indicated that GPCRs could also serve as targets for the development of new insecticides^33^; a dopamine receptor, AaDOP2, has been identified as the target for new larvicide development in *Aedes aegypti* by cell-based chemical screen^34^,^35^. Rediocides, found in a natural terpenoid-based plant extract with insecticidal properties, could act to inhibit *Drosophila* GPCR by decreasing the calcium response and thus leading to GPCR insensitivity via protein kinase C activation in HEK 293 cells^36^. Interestingly, up-regulated GPCRs have also been reported in resistant *Culex* mosquitoes^37^,^38^. Multiple gene interaction and regulation play pivotal roles in insecticide resistance^37^,^39^,^40^. Thus, the transcriptionally up-regulated gene expression of GPCRs in insecticide resistance offers a promising new avenue for research. To address the potential function of GPCRs and GPCR-related genes in the development of insecticide resistance in the mosquito, *Cx. quinquefasciatus*, we therefore characterized the expression level of a total of 115 GPCR and GPCR-related genes in a series of susceptible to highly resistant *Culex* mosquito strains. Double-stranded RNA interference (RNAi) was utilized to silence the up-regulated GPCR-related genes in the resistant mosquito strains in order to investigate their function in insecticide resistance by regulating the expression of resistance-related P450 genes. Our findings suggest that the resulting interference with GPCR and GPCR-related gene expression could provide a new opportunity for resistance prevention in mosquitoes and other insect pests.

## Results

### GPCR genes in *Cx. quinquefasciatus* genome

Based on the GPCR annotation information in the GPCRDB and the *Cx. quinquefasciatus* genome, we tested 68 GPCR genes in this study. These consisted of 52 annotated GPCR genes in the class A, rhodopsin-like family, with 15 amine receptors, 25 peptide receptors, 1 adenosine A2 receptor, and 11 opsin receptors; 4 GABA_B_ receptors; and 12 orphan GPCR genes (Table 1). Given the focus of this study is on GPCR-biological process analysis, 47 GPCR-related genes were also identified as potential GPCRs regulatory pathways in insecticide resistance regulation. The predominance of rhodopsin-like GPCRs in the *Culex* genome suggests that this cluster of GPCR genes is likely to perform a critical function in various signal transduction pathways in *Cx. quinquefasciatus*.

### Relative expression of GPCR-related genes in the *Cx. quinquefasciatus* strains

Understanding the changes in the GPCR-related gene expression in resistant and susceptible mosquito strains could shed light on the potential roles of these genes in the development of insecticide resistance in mosquitoes. In this study, we characterized a total of 115 GPCR and GPCR-related genes by examining their expression levels in both larvae and adults of five mosquito strains using qRT-PCR with 115 uniquely designed primer pairs (Table S1). The five strains exhibit different resistance profiles in response to permethrin, ranging from the most susceptible strain, S-Lab, through the intermediately resistant strains, HAmCq^G0^ and MAmCq^G0^, to the highly resistant strains, HAmCq^G8^ and MAmCq^G6^ 8. The GPCR-related gene expression profiles revealed that four GPCR and GPCR-related genes were significantly up-regulated and 11 genes were down-regulated in the highly resistant mosquitoes compared to the intermediately resistant and susceptible strains. However, the remaining 100 genes were equally expressed in all strains (Table S2). These results suggest the possibility that these up-regulated GPCR-related genes are indeed involved in permethrin resistance in *Culex* mosquitoes.

### Up-regulated GPCR genes in both larvae and adults of the resistant strains HAmCq^G8^ and MAmCq^G6^

**Figure 1:**
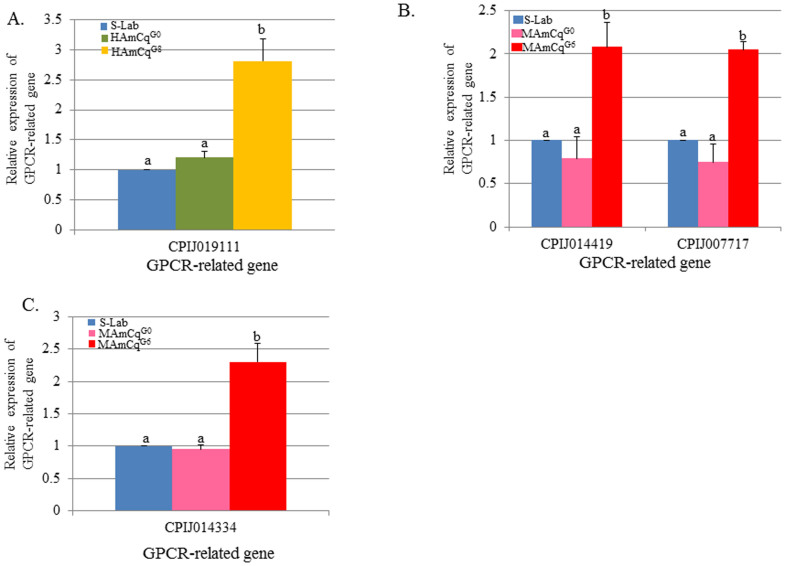
Relative expression of 4 GPCR-related genes analyzed by qRT-PCR in mosquito strains of *Cx.* *quinquefasciatus*. A. Relative expression of a GPCR-related gene, CPIJ019111, in 4^th^ instar larvae of S-Lab, HAmCq^G0^ and HAmCq^G8^. B. Relative expression of 2 GPCR-related genes, CPIJ014419 and -007717, in 4^th^ instar larvae of S-Lab, MAmCq^G0^ and MAmCq^G6^. C. Relative expression of a GPCR-related gene, CPIJ014334, in 3-day-old adult S-Lab, MAmCq^G0 ^and MAmCq^G6^. The relative gene expression shown along the Y axis is the ratio of the gene expression in each resistant strain compared with the susceptible strain. The results are shown as the mean ± S.E. There was significant difference (P ≤ 0.05) in the levels of P450 gene expression among the samples with the different alphabetic letter (i.e., a, b, or c).

We found that a GPCR-related gene (CPIJ019111, conserved hypothetical protein) was significantly up-regulated (~3-fold) in the 4^th^ instar of HAmCq^G8^ compared with S-Lab and HAmCq^G0^ (P ≤ 0.001) (Fig. 1A). A calcitonin receptor (CPIJ014419) was significantly up-regulated (~2-fold) in the 4^th^ instar of MAmCq^G6^ compared with S-Lab and MAmCq^G0^ (P = 0.002). A similar expression pattern was also found for a GPCR-related gene coding for a conserved hypothetical protein (CPIJ007717); the expression of the gene was ~2-fold higher in the 4^th^ instar of MAmCq^G6^ compared with S-Lab and MAmCq^G0^ (P ≤ 0.001) (Fig. 1B). A pteropsin gene (CPIJ014334) was expressed at a significantly higher rate, ~2.5-fold higher, in adult MAmCq^G6^ mosquitoes than in MAmCq^G0^ and S-Lab adults (P ≤ 0.001) (Fig. 1C). All these up-regulated GPCR-related genes in HAmCq^G8^ and MAmCq^G6^ adults have similar expression levels in both S-Lab and HAmCq^G0^ adults (Fig. 1). These results indicate the importance of the up-regulated GPCR-related genes in permethrin resistance following permethrin selection.

### Down-regulated GPCR genes in both larvae and adults of the resistant strains HAmCq^G8^ and MAmCq^G6^

**Figure 2:**
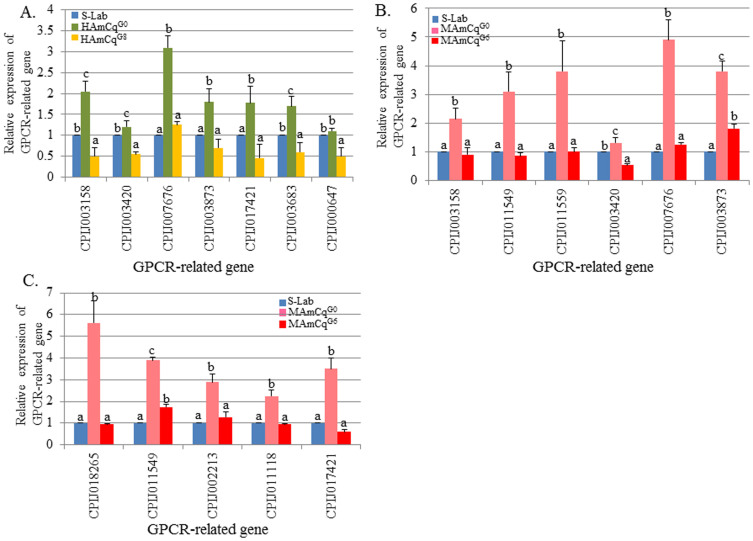
Relative expression of 11 GPCR-related genes analyzed by qRT-PCR in mosquito strains of *Cx.* *quinquefasciatus*. (A). Relative expression of 7 GPCR-related genes in 4^th^ instar larvae of HAmCq^G0^ and HAmCq^G8^. (B). Relative expression of 6 GPCR-related genes in 4^th^ instar larvae of MAmCq^G0^ and MAmCq^G6^. (C). Relative expression of 5 GPCR-related genes in 3-day-old adult MAmCq^G0^ and MAmCq^G6^. The Y-axis represents the fold change of the expression of each gene in resistant strains compared with the susceptible S-Lab strain. The results are shown as the mean ± S.E. There was significant difference (P ≤ 0.05) in the levels of P450 gene expression among the samples with the different alphabetic letter (i.e., a, b, or c).

With regard to GPCRs that were down-regulated in the permethrin selected strains using qRT-PCR, we found several GPCR and GPCR-related genes were down-regulated (≤-2-fold) in HAmCq^G8^ and MAmCq^G6^ compared with their parental strains. Seven genes were significantly down-regulated in the larvae of HAmCq^G8^ compared to the parental strain HAmCq^G0^, including CPIJ003158 (GPCR) (P = 0.003), -003873 (beta adrenergic receptor) (P = 0.028), -003683 (5-hydroxytryptamine receptor 2B) (P = 0.015), and 4 GPCR-related genes as following -003420 (P = 0.007), -007676 (P ≤ 0.001), -017421(P = 0.042), and -000647(P = 0.035) (Fig. 2A). The expression of the majority of these genes in HAmCq^G8^ was at a lower level than that observed in the susceptible S-Lab mosquitoes, even though most were expressed at higher levels in HAmCq^G0^ than in S-Lab (Fig. 2A). We also found a similar down-regulation pattern in MAmCq^G6^ larvae compared with that in MAmCq^G0^ and S-Lab larvae, with a GPCR gene (CPIJ003158) (P = 0.031), a calcitonin receptor (-011559) (P = 0.04), a beta adrenergic receptor (-003873) (0.002), and 3 GPCR-related genes as following -011549 (P = 0.018), -003420 (P = 0.01), and -007676 (P = 0.002) all having ~2-fold significantly lower expression than in MAmCq^G0^ (Fig. 2B). Most of these genes exhibited similar expression levels in MAmCq^G6^ larvae compared with those in S-Lab larvae, even though they were expressed at higher levels in MAmCq^G0^ than in S-Lab (Fig. 2B). Moreover, given the study of down-regulated genes in adult MAmCq^G6^ mosquitoes compared with MAmCq^G0^ adults, 5 genes were significantly down-regulated (≤2-fold): a GPCR gene (CPIJ018265) (P = 0.003), an allatostatin receptor (-011118) (P = 0.004), and 3 GPCR-related genes as following -011549 (P = 0.011), -002213 (P = 0.011), -017421 (P = 0.046) (Fig. 2C). Again, the expression levels of these down-regulated genes were similar in adult MAmCq^G6^ mosquitoes compared with S-Lab adults, even though all were at higher levels in MAmCq^G0^ compared with S-Lab (Fig. 2C). In summary, the GPCR gene (CPIJ003158), a beta-adrenergic receptor (-003873), and 2 GPCR-related genes (-003420, -007676) were down-regulated in the larvae of both HAmCq^G8^ and MAmCq^G6^, and CPIJ011549 was down-regulated in both larvae and adults MAmCq^G6^ mosquitoes. The GPCR-related gene (CPIJ017421) was down-regulated in both HAmCq^G8^ larvae and MAmCq^G6^ adults. The down-regulated GPCR-related genes represented in the resistant mosquito strains probably shut down after permethrin selection in order to conserve energy, which could then be used for the production of resistance-related gene expression.

### Microinjection of CPIJ019111 dsRNA of in HAmCq^G8^

**Figure 3:**
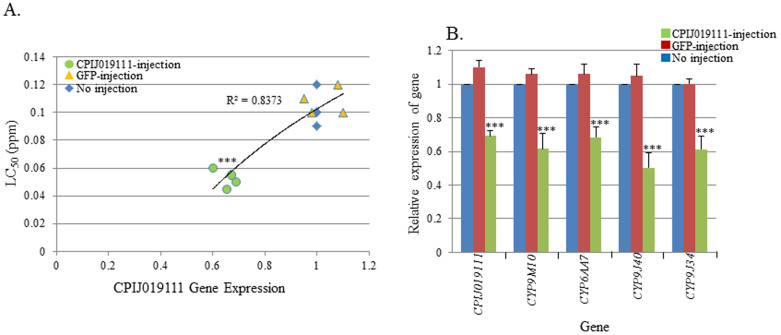
Functional study of the GPCR019111 gene in the permethrin resistant HAmCq^G8^ strain using RNAi. (A). The correlation between GPCR019111 expression and susceptibility to permethrin was determined by dsRNA knockdown of the GPCR019111 gene in HAmCq^G8^ mosquitoes compared to non-injected and GFP-injected mosquitoes. The Y-axis represents the LC_50_ of permethrin in dsRNA-injected and non-injected mosquitoes. (B). Relative expression of 4 cytochrome P450 genes in dsRNA of GPCR019111-injected, GFP-injected and non-injected mosquitoes. The relative gene expression shown along the Y-axis is the ratio of the gene expression in dsRNA-injected mosquitoes compared with that in non-injected mosquitoes. The results are shown as the mean ± S.E. Significant differences are indicated by *(P ≤ 0.05), **(P ≤ 0.01), and ***(P ≤ 0.001).

To investigate the function of the up-regulated GPCR-related genes in *Culex* mosquitoes, we injected CPIJ019111 dsRNA into HAmCq^G8^ embryos to knockdown the target gene in HAmCq^G8^ larvae. Comparing the relative gene expression and larval sensitivity to permethrin in the CPIJ019111 dsRNA injected HAmCq^G8^ larvae with those of GFP-injected or no-injected larvae revealed a strong correlation between the gene expression of CPIJ019111 and sensitivity to permethrin (R^2^ = 0.8373). Knockdown of CPIJ019111 (1.6-fold decrease in gene expression, P ≤ 0.001) significantly correlated with the decrease of the LC_50_ of permethrin in CPIJ019111 dsRNA-injected larvae compared to GFP-injected and non-injected larvae (Fig. 3A). To confirm the involvement of CPIJ019111 dsRNA in the regulation of resistance-related P450 gene expression, we tested changes in the expression of 4 P450 genes (CYP9M10, -9J34, -9J40, -6AA7) known to be involved in permethrin resistance in *Cx. quinquefasciatus*^11^,^12^ in CPIJ019111 knockdown mosquitoes. The results showed that suppression of CPIJ019111 expression also significantly decreased the expression of all four P450 genes (Fig. 3B, P ≤ 0.001).

### Microinjection of CPIJ007717 and -014419 dsRNAs into MAmCq^G6^

**Figure 4:**
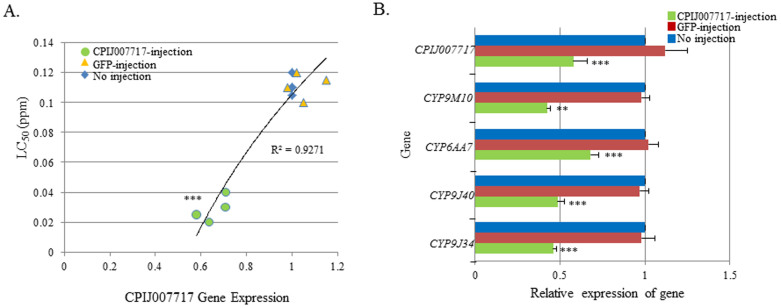
Functional study of the GPCR007717 gene in the permethrin resistant MAmCq^G6^ strain using RNAi. (A). The correlation between GPCR007717 and susceptibility to permethrin was characterized by dsRNA knockdown of the GPCR007717 gene in MAmCq^G6^ mosquitoes compared to non-injected and GFP-injected mosquitoes. The Y-axis represents the LC_50_ of permethrin in dsRNA-injected and non-injected mosquitoes. (B). Relative expression of 4 cytochrome P450 genes in dsRNA of GPCR007717-injected, GFP-injected and non-injected mosquitoes. The relative gene expression shown along the X-axis is the ratio of the gene expression in dsRNA-injected mosquitoes compared with that in non-injected ones. The results are shown as the mean ± S.E. Significant differences are indicated by *(P ≤ 0.05), **(P ≤ 0.01), and ***(P ≤ 0.001).

**Figure 5:**
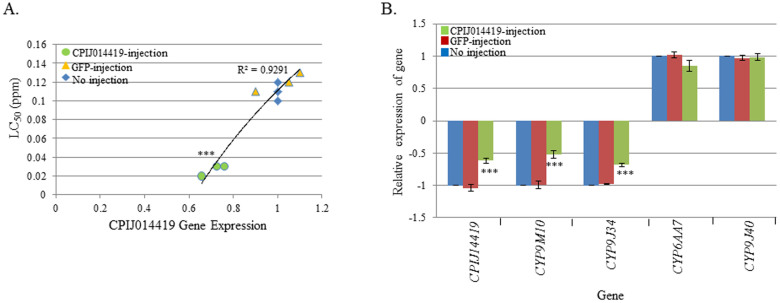
Functional study of the GPCR014419 gene in the permethrin resistant MAmCq^G6^ strain using RNAi. (A). The correlation between GPCR014419 and susceptibility to permethrin was characterized by dsRNA knockdown of the GPCR014419 gene in MAmCq^G6^ mosquitoes compared to non-injected and GFP-injected mosquitoes. The Y-axis represents the LC_50_ of permethrin in dsRNA-injected and non-injected mosquitoes. (B). Relative expression of 4 cytochrome P450 genes in dsRNA of GPCR014419-injected, GFP-injected and non-injected mosquitoes. The relative gene expression shown along the Y-axis is the ratio of the gene expression in dsRNA-injected mosquitoes compared with that in non-injected ones. The results are shown as the mean ± S.E. Significant differences are indicated by *(P ≤ 0.05), **(P ≤ 0.01), and ***(P ≤ 0.001).

Two of the up-regulated GPCR-related genes, CPIJ007717 and CPIJ014419, may play critical roles in permethrin resistance in MAmCq^G6^. Thus, CPIJ007717 and -014419 dsRNAs were each injected into early embryos of MAmCq^G6^ to investigate their function in insecticide resistance. The results revealed that knockdown of the CPIJ007717 gene (with 1.9-fold decrease in expression, P ≤ 0.001) was significantly correlated with an increased sensitivity to permethrin and decreased LC_50_ (R^2^ = 0.9271, Fig. 4A). The regulatory function of CPIJ007717 on resistance-related P450 gene expression was also confirmed, as repression of the CPIJ007717 gene significantly decreased the expression of 4 P450 genes (P = 0.003 for *CYP9M10* or P ≤ 0.001, Fig. 4B). We also found a similar result in MAmCq^G6^ larvae with dsRNA interference of CPIJ014419, where a strong correlation was observed between the expression of CPIJ014419 and sensitivity to permethrin (R^2^ = 0.9291). Decreased expression of CPIJ014419 (1.7-fold) significantly decreased the LC_50_ of permethrin in larvae of CPIJ014419 injected MAmCq^G6^ mosquitoes compared with GFP-injected and non-injected larvae (P ≤ 0.001, Fig. 5A). Examining the regulatory function of CPIJ014419 on P450 gene expression revealed that the expression two P450 genes, *CYP9M10* and *-9J34*, was significantly decreased in CPIJ014419 asRNA-injected mosquitoes (P ≤ 0.001, Fig. 5B). However, two other P450 genes, *CYP6AA7* and *-9J40*, exhibited no change in gene expression following the CPIJ014419 expression repression (Fig. 5B). These results confirmed the importance of up-regulated GPCR-related genes in permethrin resistance and the involvement in the regulation of some of insecticide resistance-related P450 genes in MAmCq^G6^ mosquitoes.

### Microinjection of CPIJ014334 dsRNA into adults of MAmCq^G6^

**Figure 6:**
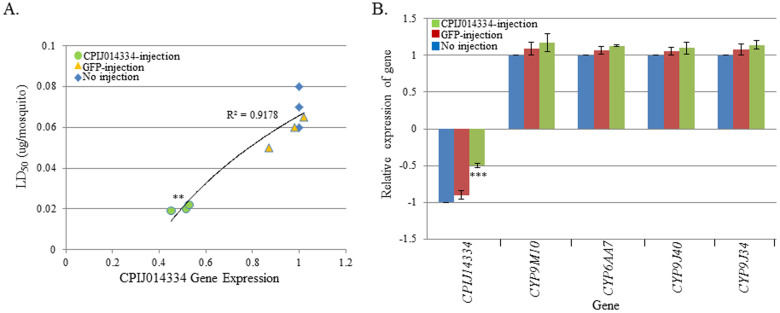
Functional study of the GPCR014334 gene in female adults of the permethrin resistance strain MAmCq^G6^ using RNAi. (A). The correlation between GPCR014334 and susceptibility to permethrin was characterized by dsRNA knockdown of the GPCR014334 gene in MAmCq^G6 ^mosquitoes compared to non-injected and GFP-injected mosquitoes. The Y-axis represents the LC_50_ of permethrin in dsRNA-injected and non-injected mosquitoes. (B). Relative expression of 4 cytochrome P450 genes in dsRNA of GPCR014334-injected, GFP-injected and non-injected mosquitoes. The relative gene expression shown along the Y-axis is the ratio of the gene expression in dsRNA-injected mosquitoes compared with that in non-injected mosquitoes. The results are shown as the mean ± S.E. Significant differences are indicated by *(P ≤ 0.05), **(P ≤ 0.01), and ***(P ≤ 0.001).

As only one GPCR-related gene, CPIJ014334, was found to be up-regulated in adults of the highly resistant mosquito strain, MAmCq^G6^, we injected CPIJ014334 dsRNA into adult MAmCq^G6^ mosquitoes. A 2-fold decrease in the expression of CPIJ014334 gene was significantly correlated with a decreased LD_50_ of permethrin in 3-day post-injection female mosquitoes compared to their non-injected and GFP-injected counterparts (R^2^ = 0.9178, P = 0.008, Fig. 6A). However, testing the expression of four P450 genes in CPIJ014334 knockdown mosquitoes revealed no changes in the expression of the P450 genes (Fig. 6B), indicating that the CPIJ014334 gene may perform a regulatory function on the expression of another resistance-related gene in adult MAmCq^G6^ mosquitoes.

## Discussion

Given the critical function of GPCRs in insect development, reproduction, and metabolism, it is not surprising that GPCRs have been characterized in a wide range of insect species, with 276 GPCRs being identified in *Anopheles gambiae*^41^, 135 non-sensory and opsin GPCRs in *Aedes aegypti*^33^, ~70 neurohormone GPCRs in the red flour beetle^42^,^43^, 56 neurohormone receptor genes in *Apis mellifera* and 69 in *Drosophila* *melanogaster*^30^, as well as 107 GPCRs in the *Pediculus humanus* whole genome^44^. *Cx. quinquefasciatus* genome sequencing allowed us to select 68 annotated GPCR genes and 47 GPCR biological processes that are known to be pathway-related genes for inclusion in the current study. Most of the GPCRs in *Cx. quinquefasciatus* belong to class A (Rhodopsin-like GPCR), which comprises the largest family of GPCRs and could be transducers that bind a striking and extraordinary range of ligands, including light, peptides, lipids, and nucleotides, in both vertebrates and invertebrates^45^, indicating the importance of the rho-like GPCRs in *Culex* mosquitoes.

Our previous studies demonstrated that GPCR and the GPCR-regulatory pathway may be involved in the development of insecticide resistance in *Cx. quinquefasciatus*^37^. Moreover, GPCRs have also been identified as a promising new target for future insecticide development^31^,^41^. Thus, a better understanding of the precise role of GPCR and GPCR-related genes in insecticide resistance will support the development of innovative strategies to control mosquitoes and mosquito-borne diseases. To adapt to xenobiotic exposure, insects employ a coordinated transcriptional response, an example of which is gene up-regulation in insecticide resistant insects. Thus, we expected that GPCR-related genes, which may play a role in permethrin resistance, would be up-regulated in resistant mosquitoes following permethrin selection. In the research reported here, we have, for the first time, examined the expression profiles of 115 GPCR and GPCR-related genes in larvae and adult *Cx. quinquefasciatus* mosquitoes by comparing the different expression levels among two highly resistant mosquito strains, HAmCq^G8^ and MAmCq^G6^, two intermediately resistant strains, HAmCq^G0^ and MAmCq^G0^, and a susceptible strain, S-Lab. Our results reveal a dynamic change in the expression of these 115 GPCR-related genes between the permethrin resistant and susceptible strains. Interestingly, the genes that were up-regulated following permethrin selection in the mosquito larval stage were not found in the adult stage, and vice versa. Additionally, comparing the up-regulated GPCR-related genes in the two highly resistant mosquito strains revealed different gene sets to be involved in their response to permethrin selection, indicating the possibility of different regulation pathways for the GPCRs in different mosquito strains.

Our study also revealed the existence of down-regulated GPCR expression following permethrin selection in *Cx. quinquefasciatus*. Although the mechanisms by which down-regulated GPCRs could contribute to insecticide resistance are still unclear, a number of mechanisms to explain this effect have been proposed in other insects: 1) down-regulation of the odorant receptor gene (putative GPCR) after blood feeding in *A. gambiae* could modify the organism's odorant response profile^46^; 2) down-regulation of GPCR genes was thought likely to be a response to low-intensity malarial parasites in *A. gambiae*^47^; and 3) down-regulated GPCR (PBAN-receptor) in the 4^th^ instar to 1^st^ and 2^nd^ pupa of *Ae. aegypti* compared to other stages corresponded to the up-regulation of another GPCR (diapause hormone receptor)^48^. The evidence and hypotheses reported in these studies all suggest reasonable explanations for the potential function of down-regulated GPCRs corresponding to different biological pathways in insects. Taken together, the findings of the current study may indicate that the down-regulated GPCR and GPCR-related genes were involved in the response to permethrin selection by homeostatically reacting to balance the up-regulated GPCR-related gene expression.

The use of the RNAi technique for this functional study of up-regulated GPCR-related genes in highly resistant mosquitoes provides a powerful tool for silencing gene expression post-transcriptionally^49^,^50^. For example, Bai et al. used the RNAi technique to determine the precise functions of non-sensory GPCR genes in the red flour beetle, *Tribolium castaneum*, including 6 GPCRs that had critical effects on larval and pupal molting and mortality^31^. In the current study, we used the RNAi method to repress the GPCR gene expression in resistant mosquitoes. Our findings revealed that knockdown of the up-regulated GPCR-related genes (CPIJ019111,-007717, -014419, -14413) was strong correlated with decreased resistance to permethrin, signifying the important function of these GPCR-related genes in the permethrin resistance of *Cx. quinquefasciatus*.

Cytochrome P450 gene up-regulation is known to be involved in insecticide resistance in insects^11^,^51^,^52^, but the regulatory mechanisms of P450 up-regulation are still unclear. GPCRs play essential roles in switching on chemical signals in response to environmental stimuli and in triggering several specific downstream signaling pathways with targets in the cell membrane, cytoplasm, and nucleus^53^,^54^. It has also been shown that GPCRs could be regulatory factors for resistance-related P450 gene regulation in the housefly^15^. Taken together, we think it likely that the regulatory function of these up-regulated GPCR genes in insecticide resistance is also linked to the regulation pathway of resistance-related P450 gene expression. The results demonstrated the decreased expression of P450 genes following the knockdown of GPCR-related genes (CPIJ019111, -007717, and -14419), suggesting that these up-regulated GPCR-related genes could be critical for P450 gene expression. However, knockdown of the GPCR-related gene (CPIJ014413) in adult MAmCq^G6^ mosquitoes lowered resistance to permethrin but had no effect on the expression of any of these four P450 genes, suggesting that it either regulates the expression of another resistance-related P450 gene besides that is not one of the four genes tested or is involved in a completely separate regulatory pathway for resistance-related gene expression. The mechanisms by which the GPCR regulation pathway in permethrin resistant *Culex* mosquitoes appears to operate also suggest that the importance of the up-regulation of GPCR-related gene expression lies in its ability to regulate the resistance-related gene expression beyond the signal stimulation that caused the original cell response.

This study has shed new light on the potential function of GPCR and GPCR-related genes in insecticide resistance and their regulatory function on resistance-related P450 gene expression. However, the entire regulatory pathway remains largely unclear. Thus, future research will focus on the downstream factors in the GPCR regulatory pathway in order to characterize their involvement in insecticide resistance and, consequently, support the effort to identify new insecticide targets and strategies with which to control mosquitoes and mosquito-borne diseases, as well as helping to build a better understanding of the regulatory pathway of insecticide detoxification and evolutionary insecticide selection in mosquitoes.

## Methods

### Mosquito strains

Five mosquito strains of *Cx. quinquefasciatus* were used in this study. Two field strains, HAmCq^G0^ and MAmCq^G0^, were collected from Madison County and Mobile County, AL, respectively, which are located more than 600 km apart; HAmCq^G8^ is the eighth generation of permethrin-selection HAmCq^G0^ offspring; MAmCq^G6^ is the sixth generation of permethrin-selected MAmCq^G0^ offspring^8^,^12^; and S-Lab is an insecticide susceptible strain kindly provided by Dr. Laura Harrington (Cornell University, Ithaca, NY). All the mosquitoes were reared at 25 ± 2°C under a photoperiod of 12:12 (L:D) h (insectary conditions) and fed blood samples from horses (Large Animal Teaching Hospital, College of Veterinary Medicine, Auburn University, Auburn, AL).

### RNA extraction and cDNA preparation

Total RNAs were extracted from 4^th^ instar larvae and 3 day-old adults (collected prior to blood feeding) of each mosquito strain using the acidic guanidine thiocyanate-phenol-chloroform method^14^. The DNA was removed from total RNA (5 ug) using TURBO DNA-free (Ambion) following the manufacturer's instructions. cDNA was synthesized using the DNA-free RNA as a template and the Transcriptor First Strand cDNA Synthesis kit (Roche), following the manufacturer's instructions. The quantity of cDNA produced was measured by a spectrophotometer prior to qRT-PCR. Each experiment was repeated more than 3 times, with independent RNA preparation and cDNA synthesis.

### Quantitative real-time PCR (qRT-PCR)

According to the database maintained by GPCRDB (http://www.gpcr.org/7tm/search) and GPCR biological processes related GPCR genes (https://www.vectorbase.org/search), 68 GPCR genes and 47 GPCR-related gene full length were exported from the *Cx. quinquefasciatus* whole genome sequence database (https://www.vectorbase.org/organisms/culex-quinquefasciatus). The qRT-PCR was performed with the FastStart Universal SYBR Green master mix Kit (Roche) and ABI 7500 Real Time PCR system (Applied Biosystems). Each qRT-PCR reaction was run in 3 replicates, for a total reaction volume of 25 μl, and contained SYBR Green master mix, specific primer pairs of GPCR, and GPCR-related genes at a final concentration of 3–5 μM that were designed based on each of the GPCR-related gene sequences (HYPERLINK “http://cquinquefasciatus.vectorbase.org/”), as listed in Table S1 with the accession number for each GPCR-related gene, as well as a 1 µg cDNA template from each mosquito sample. A ‘no-template’ negative control was also performed for each. The reaction cycle consisted of a melting step of 50°C for 2 min, then 95°C for 10 min, followed by 40 cycles of 95°C for 15 sec and 60°C for 1 min. Specificity of the GPCR-related gene PCR reactions was assessed by a melting curve analysis using Dissociation Curves software^55^. Relative expression levels of GPCR-related genes were calculated by the 2^−ΔΔCT^ method using SDS RQ software^56^. The 18S ribosomal RNA (rRNA) gene served as an endogenous control^11^,^12^. Each experiment was repeated 3 times with 3 independently isolated RNA mosquito samples. The statistical significance of the gene expression was calculated using a Student's t-test for all 2-sample comparisons and a one-way analysis of variance (ANOVA) for multiple sample comparisons using Statistical Package for the Social Sciences (SPSS) software with both Least Significant Difference (LSD) and Tukey tests to analyze the significance of means. A value of P ≤ 0.05 was considered statistically significant. Significant up-regulation or down-regulation was determined using a cut-off value of a “2-fold change in expression”^10^.

### Double-stranded RNA (dsRNA) synthesis

The synthesis of the dsRNA (~200 bp-650 bp based on the full length of GPCR-related genes) was performed *in vitro* using the MEGAscrip T7 High Yield Transcription kit (Ambion). The specific primers designed with the T7 promoter to amplify the up-regulated GPCR-related genes (CPIJ019111, -014419, -007717, and -014334) are listed in Table S3. For dsRNA purification, a phenol/chloroform extraction was followed by ethanol precipitation. A dsRNA of a green fluorescent protein gene was generated that was complementary to a pMW1650 plasmid (a generous gift from Dr. Zhiyong Xi, Department of Microbiology and Molecular Genetics, Michigan State University) and served as a negative control for the dsRNA injection. Non-injected mosquitoes from the same strain reared under the same rearing conditions were used for calibration.

### Adult injection with dsRNA of GPCR-related genes

To investigate the precise role of the up-regulated GPCR-related genes in resistant mosquitoes, we used the dsRNA interference (RNAi) technique to knockdown an adult up-regulated GPCR-related gene (CPIJ014334) in the resistant strain MAmCq^G6^ based on the mosquito adult injection method^57^,^58^. The microinjection glass needle was pulled from a borosilicate glass capillary tube (1 mm OD x 0.58 ID mm, 100 mm length, World Precision Instruments, Inc.) using a Model P-97 Flaming/Brown micropipette puller (Sutter Instrument Co.), following the program: Heat 454, Vel 120, Time 125, and Pull 30. Forceps were used to open the tip of the needle and the Nanoject II injector (Drummond Scientific Company) to draw dsRNA into the glass needle for injection. Approximately 138 nl of dsRNA (with a dsRNA concentration of 3.5 µg/µl in distilled water) was injected into the thorax of CO_2_-anesthetized 1-d-old female mosquitoes using the Nanoject II injector and Drosophila CO_2_ Fly Pads (Tritech Research, Inc.). To determine if there is any non-specific effect of dsRNA injection, we injected dsRNA of the GFP gene as a negative control and non-injected mosquitoes were used for the calibration, as noted above. Both injected female mosquitoes (~100 individuals) and an equal number of non-injected mosquitoes were transferred and provided with 10% sugar solution under insectary conditions (25 ± 2°C and a photoperiod of 12:12 (L:D) h) for 3 days. The involvement of the gene in resistance against permethrin was investigated by using a topical application assay with a series concentration of permethrin designed to result in >0 and <100% mortality for each of the five strains, as previously described^8^. Total RNA was then extracted from the dsRNA-injected and non-injected female mosquitoes and the relative expression of CPIJ014334 determined using qRT-PCR with primer pairs of this gene (Table S3).

Since the GPCR-related genes are up-stream and function to regulate the downstream gene expression, we considered it possible that they could be involved in resistance-related P450 gene expression. We therefore chose 4 P450 genes (*CYP9M10*, *-9J34*, *-9J40*, and *-6AA7*) that are known to play very important roles in the permethrin resistance of *Cx. quinquefasciatus*^11^,^12^, and tested their gene expression to identify the regulatory function of CPIJ014334. Each experiment was repeated at least 3 times with independent microinjection and RNA isolation.

### Embryo injection with dsRNA of up-regulated GPCR-related genes

According to the literature, *Drosophila* embryo injection with dsRNA^59^, *Aedes aegypti* embryo injection methods and mosquito transgenic methods^60^ have all been used to examine the function of up-regulated GPCR genes (CPIJ019111, -7717, -14419). In this study, larvae of the resistant mosquito strains HAmCq^G8^ and MAmCq^G6^ were tested by injecting the dsRNA of the target gene into mosquito embryos. Around 1000 fresh grey embryos were collected from HAmCq^G8^ and MAmCq^G6^ mosquito cages in which the mosquitoes had been fed with blood three days earlier and reared under insectary conditions. Around 120 embryos were arranged on each piece of paper filter. Embryos were allowed to dry for 2–3 min and transferred to a microscope cover slide (VWR Scientific Products) using double sided tape, and then covered with Halocarbon 700 oil (Halocarbon Products Corp.) for protection against dehydration. As before, a glass capillary tube (borosil 1.0 mm OD × 0.5 mm ID/Fiber, 100 mm length, FHC, Inc) was pulled using the Model P-97 Flaming/Brown micropipette puller (Sutter Instrument Co.), following the program: Heat 525, Vel 50, Time 250, and Pull 50. The glass needle tip was opened using the Model BV-10, K. T. Brown Type micropipette beveler (Sutter Instrument Co.). We injected 0.2–0.5 nl of dsRNA (3.5 ug/ul) into the embryo posterior horizontally using the Picospritzer III injector system (Parker Instrumentation) under the Nikon Eclipse TS100 microscope (Nikon Instruments). Injected embryos were individually transferred by a needle onto another wet filter paper, and then water was used to move all the injected embryos into a clean water container. The water container was kept under insectary conditions for 3–4 days. For each gene, about 500 embryos were injected with the corresponding dsRNA: the CPIJ019111 gene in HAmCq^G8^, the CPIJ014419 and -007717 genes in MAmCq^G6^, and the GFP gene in both strains. Hatched 2^nd^ instar larvae were separated into two groups: one group was tested for larva bioassay with a series concentration of permethrin designed to result in >0 and <100% mortality, as previously described^8^, and the other group was prepared for gene expression identification of the up-regulated GPCR-related gene and of permethrin resistance-related P450 genes (*CYP9M10*, *-9J34*, *-9J40*, and *-6AA7*) using qRT-PCR. Each experiment was repeated at least 3 times with independent microinjection and RNA isolation.

## Author Contributions

Performed the experiments: T.L., L.L. Analyzed the data: N.L., T.L., L.L. L.Z. Contributed reagents/materials/analysis tools: N.L. Wrote the paper: N.L., T.L., L.L. L.Z.

## Supplementary Material

Supplementary Information Table S1-S3

## Figures and Tables

**Table 1:** Summary of GPCRs identified in *Culex quinquefasciatus*

Class A	Amines	Dopamine	3
Adrenergic			4
Muscarinic acetylcholine			1
Octopamine			4
5-hydroxytryptamine			2
FMRFamide			1
	Peptides	Neuromedin U like	1
Myokinin			1
Sulfakinin			2
Endothelin B			1
Cardioacceleratory peptide			2
Cholecystokinin CCK			1
Neuropeptide Y			2
Calcitonin			3
Somatostatin			2
Substance P			1
Pyrokinin			2
Cap2b			1
Diuretic hormone			1
Corazonin			1
Allatostatin			3
Gonadotropin releasing hormone			1
	Opsin		11
		Adenosine A2	1
Class C	GABAB		4
Orphan			12
Other GPCR-related genes (by biological process relationship)			47
*Total*			*115*
