# Supplementary material for: Role of G-protein-coupled Receptor-related Genes in Insecticide Resistance of the Mosquito, Culex quinquefasciatus
Source: Sci Rep. 2014 Sep 29;4:6474. doi: 10.1038/srep06474 (PMC4178296; doi:10.1038/srep06474)
Supplement: Supplementary Information — Table S1-S3 [file srep06474-s1.pdf]

Role of G-protein-coupled Receptor-related Genes in Insecticide Resistance of the Mosquito, *Culex quinquefasciatus*

Ting Li, Lena Liu, Nannan Liu*

Supplement Information

Table S1. Oligonucleotide primers used in qRT-PCR and PCR reactions

Transcript ID[a]	Accession No.	Gene[b]	Forward Primer (5′ to 3′)[c]	Reverse Primer (5′ to 3′)[c]
CPIJ013660	XM_001864055	octopamine receptor oamb	CGAACCCACATGGGCAAGAACTTT	TATACAGAACCCGGACAGCAGGTT
CPIJ015294	XM_001865540	dopamine receptor, invertebrate	TCTACAAGGTGAACAAGGCGTCCA	ATGATGTTGCACGTGAAGAAGGGC
CPIJ019015	XM_001869366	octopamine receptor oamb	TCAGTATGGAAATCACACGCCGGT	TCGTCGAAACCGGGAAAGGAAGAT
CPIJ018698	XM_001869052	ribosomal protein S6	ACCACATTCCGGATTGCTGATTGC	CAGGATGCACAGTTTGCGGATGTT
CPIJ001044	XM_001842661	muscarinic acetylcholine receptor gar-3	CGCTGTTGTGTGATTTGTGGCTCT	TTGGTTCGCCAGCTGCGATATTTG
CPIJ008330	XM_001849662	conserved hypothetical protein	TGGTCAGCATGATCCCGGACATTT	AATCGTGTAAACCGAGTCCTGGCT
CPIJ011883	XM_001863239	conserved hypothetical protein	AGCTGCAGAACGTCACCAACTACT	TGAACCCAAACGGCCAGTAACCTA
CPIJ003188	XM_001844724	g-protein coupled receptor	ATACCAACTTCCGGGATGCCTTCA	ACGGGACGATTTCTGTTGTGGAGA

CPIJ016039	XM_001866016	conserved hypothetical protein	CAAGAACTTCAAGCACAGGCCGTT	TGAAACGACCGTAATTTCCGCTGC
CPIJ015747	XM_001866028	5-hydroxytryptamine receptor 1	GCAACGCGACATTCCCAAGTTGTA	ACCACACTGAGTGCGTCTGAAGAT
CPIJ014487	XM_001864681	beta adrenergic receptor	CATCTTTGGCAACCTGCTGGTCAT	TGACAGCGTACGGCTTGTTCACTA
CPIJ013218	XM_001863307	tyramine/octopamine receptor	TAAGACTAATCCAACCAGCGCCGT	GCGGAACGATTGTTTCATGCGACT
CPIJ003421	XM_001845139	conserved hypothetical protein	ATTGCCAAGAACCTGATTGCGAGC	AGCAAACAGGTGAAGAATGCGAGC
CPIJ014065	XM_001864128	neuromedin-U receptor 1	TCGAGCTGTCGACGTTTGTGTTCT	CGAGCGCAACTTCAACCCAATCAA
CPIJ005574	XM_001847763	sulfakinin receptor	TACACATACGTCAGCAGCAACGGA	AGCAACAGCACAGACAGAGGAAGA
CPIJ016281	XM_001866703	sulfakinin receptor	GCGCACCATCACCAACCTATTTCT	TGCCACTGAAGAGGCTTGCAGATA
CPIJ013069	XM_001863157	neuropeptide Y receptor type 2	ACGTGGTCTTCCGGAATAAAGCCA	CGTGTGACAAATGAGCTTGCCGAA
CPIJ007187	XM_001849241	FMRFamide receptor	TGTTCATCTACCTGCTGCCGTTCA	GCAGAAACACAATGACCACGCAGA
CPIJ006984	XM_	g-protein coupled receptor	CGAAGCTGAACTTGACGTTGGACA	TTTGCGGTTGTTGCTGTACTGCTC

	001848696			
CPIJ018265	XM_001869016	g-protein coupled receptor	GCCTAGAGGCGATACAAATTGTTG	CACCATGTGGCGCTGTTGTTGATT
CPIJ014670	XM_001865310	endothelin B receptor	AGAATACCAGTGTAGCGGTGGCAA	CCAAACCAGCAACACCACGATCAA
CPIJ012071	XM_001862415	myokinin receptor	TGTGTGCGGACGGAGAGGATAAAT	ATCCATATCACCAGCGAGTTGCCT
CPIJ018504	XM_001868775	neuropeptide Y receptor	ACAGCTGCTACAACCCGATCATCT	GCTGGTGCTAACATCACTTCGCTT
CPIJ010469	XM_001854800	somatostatin receptor type 5	ACTCGGTGTACGAGATTTGCCCAT	TATCAGAAAGCACTGGTCCGCGAT
CPIJ011191	XM_001859633	somatostatin receptor	TATGCCGACCACGTACACACTGTT	TGTGCATCGTGGCAATCAAGAACG
CPIJ003158	XM_001844694	G protein-coupled receptor	ACCGCGAAACCTCCTCACAGTAAT	CTGATTGCCGTCAGCGGCATATTT
CPIJ011716	XM_001862544	g-protein coupled receptor	TTGCCCTGGGTATCAATCTTCCGT	TGGCGAACAGAAAGTACCAGCTCT
CPIJ016695	XM_001867185	g-protein coupled receptor	TTGCCCTGGGTATCAATCTTCCGT	TGGCGAACAGAAAGTACCAGCTCT
CPIJ006269	XM_001847618	cardioacceleratory peptide receptor	GCATCATTCCAAAGGCGAAGGTGA	TTGGTCTGCGTTTCCGGAATTTGG

CPIJ007712	XM_001849183	leucine-rich transmembrane protein	GTTTCGGTTGGTTGCCCATTCTGT	ATTGTTTCGTGGCCACTTCGGATG
CPIJ011619	XM_001861641	leucine-rich transmembrane protein	AGCCGGAAATAGCATAAGCTCCCT	TGAAGGCGTCCTGGTGGATAAAGT
CPIJ001199	XM_001842816	gonadotropin-releasing hormone receptor	TCTGTCCGTGGTGATTACGCTGTT	GCGGAATCATGATGAATGCCACCA
CPIJ005000	XM_001846589	short wavelength sensitive opsin	TGCTGGCGTTGATCTACTTTGTGC	AAGGCATTCACCAGAAACATCGGC
CPIJ009246	XM_001851105	ultraviolet wavelength sensitive opsin	AATCGTAAGCCACGTCGTGAACCA	CCACGAAGCCACAAACAGGAAACA
CPIJ013408	XM_001863511	short wavelength sensitive opsin	CAAGCATCGCTGTTTGTGCTGCTA	AACACCATTGGAATCGCGTAAGCC
CPIJ004067	XM_001845645	opsin (long wavelength sensitive)	TCGCACTCGTTACGATCTCGCTTT	ACTTGGGATGACTGATGCCGTACA
CPIJ012052	XM_001862130	long wavelength sensitive opsin	TCAAGATCCTGATGGTGTGGGCTT	ACACGAAGATGGCGTAGACGATGA
CPIJ011571	XM_001862163	long wavelength sensitive opsin	TCAACAGACCAGCGCTGAGATCAA	TGATTCCGTACACGATCGGGTTGT
CPIJ011573	XM_001862165	long wavelength sensitive opsin	ATGTGCCAGGTTTACGCCATGTTG	GAGCAGCGCATTGTTGAAGGTCAT
CPIJ011574	XM_00186	long wavelength sensitive opsin	TCTACTTTGCGCCTCTGCTGATGA	CAGCTTCATTTCGGTGCTGGTGTT

	2166			
CPIJ011983	XM_001862303	conserved hypothetical protein	GAGCCTGCCCAACTTTAAGCCAAA	ATGATGCAGAACGGCAACCAGAAC
CPIJ013056	XM_001863451	long wavelength sensitive opsin	CGATGGTGCAGATCTTTGGCGTTT	CAGTGGCGCAAAGTACACGAACAT
CPIJ011419	XM_001861603	unknown wavelength sensitive opsin	TGTTAACTCCGCTGGGTTCGATGA	ACTGCGAGTGGTTCATCTCCTGTT
CPIJ014334	XM_001864516	pteropsin	TCATCCTGCTGAACCTGGTGTGTT	TATGCCCAGCAGGGACATGAAGAA
CPIJ015116	XM_001865344	wd-repeat protein	CCAGCTGGTTCTGCTGCATTTGAT	TGTCCACACCCGTGAACAGAATGT
CPIJ000143	XM_001841761	g-protein coupled receptor	GTGCTGACAAGACGTGAAATGCGT	ACACTGCGTAGGGCACATAATCCA
CPIJ006507	XM_001848139	g-protein coupled receptor	ACTTCACCCAGATACTGCACACCA	TCGTGAAGACAAAGTACGTCGGCA
CPIJ011547	XM_001861835	conserved hypothetical protein	AGCTCCAGTTGGGAATGAACGAGA	ACGCTGATGATCTTGCTGTTTGCG
CPIJ011549	XM_001861837	conserved hypothetical protein	AGTGTTCCCACATGACGAGCTTCT	TCCAAACAAGGACAACAAGCAGCC
CPIJ017258	XM_001867508	neural-cadherin	ATCATGCCACAGATTCCTGGACCT	GACATCCCGCAATTGTTCCGTTGT
CPIJ0	XM_	calcitonin receptor	TCTGCTGCACTATCTGATGCTGGT	AGTTGTTCCGGAAGGTTGCGTAGA

11559	001862151			
CPIJ014419	XM_001864861	calcitonin receptor	GCGAAACTGAACGAATCCGTCGAA	TGGTGTCGAATCCGAAGACGAACT
CPIJ009749	XM_001851012	calcitonin receptor	ACAGCACTGGAATTTGCCTTGTGG	ACCGCGAATGGAGAGGTAAACACT
CPIJ008822	XM_001850659	conserved hypothetical protein	TAGAATGCTCCTGGATGCGGGAAT	AGCTTCGTGATCAGGACCCACATT
CPIJ013929	XM_001864308	conserved hypothetical protein	TGAGGCCGCATCGGTTACTATGAA	TACACGGAATGAAGTCCCAACCCA
CPIJ008641	XM_001850157	gamma-aminobutyric acid type B receptor	TGCAGGAGAACGAGTACTGCCAAT	ACATACCGACGTGTTTGGAGTCGT
CPIJ002466	XM_001844023	gamma-aminobutyric acid type B receptor	GTTGCTTCTGTTGGACGCTTGTGT	GCGTACAACGTTCCAAGCCAACTT
CPIJ008111	XM_001849791	conserved hypothetical protein	AGAAGCAGTGTTGCTGGGTTTGTG	TCGATCGGGATTTGGAGGCATTCT
CPIJ016970	XM_001867057	uridine cytidine kinase i	GAACAGTTTCGTCATTGCGGTGGT	GCGATGGCGGTTATCAAGCGATTT
CPIJ007717	XM_001849188	conserved hypothetical protein	TTCGTTGTATGGCTGGTGGCACTT	TCATCGGAATGAAGAAGGAGCCCA
CPIJ014671	XM_001865311	substance P receptor	TGACGGCCATCGTATTCCCAAAGA	ACGGTCGTTCCTTGTAGTTTCGGT

CPIJ000410	XM_001842028	protein smoothened precursor	AGCGATTGGGAAGATCGGAAAGGA	ATCGTAATCGTCAACACCAGCGGA
CPIJ011757	XM_001861922	5HT-dro2A receptor	ACTCTCCACCATTTCTAACGGCCA	TCCTCGGCGAAGCTTGTAACTTGA
CPIJ020007	XM_001870341	conserved hypothetical protein	TACTGTTCCTGAGAAATCGCCGCT	CACGCCCAACACAAACGAGAAGTT
CPIJ011105	XM_001861425	pyrokinin receptor	TATCGAGTGCAAGCGATCCTCCAA	GTGCTCCATTGTGAGGCAACCAAA
CPIJ011106	XM_001861426	pyrokinin receptor	GTTCAATCAGGTGGCCTGCATCAT	TTCGAACTTGCCGAAATGGGTGAC
CPIJ009459	XM_001851355	conserved hypothetical protein	TCGCCTTGTCGTACCTGTTTGGAT	GGTCGTTTGTGAGCAGGCATTTCA
CPIJ019111	XM_001869507	conserved hypothetical protein	ACGATCGAGGCAGAACAATCACGA	TGGAGAAGTCGAACAGCGGTTTCT
CPIJ000002	XM_001841620	conserved hypothetical protein	TGATGACCCTAGCTTTCCTGCCAA	TTCCGGACTGGCTTCATTAAGGGT
CPIJ018293	XM_001868397	Cap2b receptor	CGGCGACTCATGATGCTTCTTGTT	TTGATCAGCTCCATCAGGTTGGCT
CPIJ015979	XM_001866120	conserved hypothetical protein	ACCAAACGACGACGACGATGAAGA	GGATGTGATGGTTGCCAATGTGGT
CPIJ019916	XM_00187	conserved hypothetical protein	TGCTAGCCATCTTCACAGTGGTCA	TTGTTGAAGTGGGTGTTGCTGCTG

	0156			
CPIJ014753	XM_001865118	conserved hypothetical protein	CCAGAGCAAAGAGGAGCAAGTCAA	TCGAGATGAACGTCAACGTGAGCA
CPIJ002213	XM_001843830	Odorant receptor 94b	ACCTGCTTATTTCACGTTGGCAGC	TCAAACGCCTGCAGTGTGTGAAAG
CPIJ003422	XM_001845140	conserved hypothetical protein	TGGTGCCCATTGTCATCCTCAAGA	ACCCATACTTCGGGTGGTGAGTTT
CPIJ003420	XM_001845138	conserved hypothetical protein	ACGGCATCAACAAATCTGAGCAGC	GTTGTGGCGTTGATGAAGCAACCT
CPIJ013535	XM_001863623	GABA-B receptor	TGTGGGATAGTGGTGGCATTAGCA	TGGCTTGGCATACCTTGGGATAGT
CPIJ013536	XM_001863624	GABA receptor	TTATGTTACGGCGCATCTAGCCCT	ATGAACACTTCTTCGGCTTGCTGG
CPIJ016679	XM_001866877	conserved hypothetical protein	TGGCGGTGTACTCACAAGAGGTTT	ATGTTCCCGAGCAGCGATACGATT
CPIJ011717	XM_001862545	g-protein coupled receptor	ATGACTTTGATACCGCTGTGGACG	ATCAGCGTACGCCAGATTCACGAT
CPIJ016293	XM_001866977	conserved hypothetical protein	TTCCGGTCCAACACGAACCTCAA	TTTCACGTCCCGCTCCTTGTACTT
CPIJ007715	XM_001849186	G protein-coupled receptor	ATCTTCCCACAATCCTCTGCTCGT	TCAGTGAAGCTGCCACGTTTTGTA
CPIJ0	XM_	G protein-coupled	TGGCTTGGGTTATCTGGGACGAAA	TCGAGCCGAACTCAGAAACTCGTA

07716	001849187	receptor		
CPIJ018158	XM_001868532	frizzled-3 precursor	TCCGGAAGCGTCGAGAAGAACTTT	AGTTGTGGTGATGGTGTTGTTGCG
CPIJ007681	XM_001849511	frizzled	TGATCGATTCGTCTCGGTTCAGGT	CCTGCGTAATCGTCGATAGCATCT
CPIJ007676	XM_001849506	frizzled precursor	TCTTCATGGCCCTGTACTTCTGCT	AATCCTTCAACTTTGCCCAGTGCC
CPIJ007677	XM_001849507	frizzled precursor	CTTCTTCAGCGGCCTGTTCATTCT	AGAAGATCTTGCACATCTGGCGGT
CPIJ007682	XM_001849512	frizzled precursor	CTTCTTCAGCGGTCTGTTCATCCT	CACCATCTGGGCGTTGTATTTGGT
CPIJ008820	XM_001850657	diuretic hormone receptor	CACGCAGAAACCGGAAGAAGGTTT	TCGACGGATCCAAAGCCGTGTAAA
CPIJ000651	XM_001842362	dopamine receptor	GACGTTCGCCGGTGTCAATGATTT	TGAGCGGATCCTTGATGTGGATGT
CPIJ016678	XM_001866876	corazonin receptor	TTCTTCGGGATGTCCAACAGCCTA	TGTTGCTTGTACTGATTGTGCCGC
CPIJ019566	XM_001869764	G-protein coupled receptor	TGGTGATAGCGTTCTTCCTGTGCT	TGTACGTCAGGATTTCGAACGCGA
CPIJ011881	XM_001863237	conserved hypothetical protein	AACCTTCAATTCCCGGAGTGACCT	TGCAGAACAGCACATCCAGACAGA

CPIJ016282	XM_001866704	CCK-like GPCR	TCATCAACACGATGGCCCTGTTCT	AAGCACCGAAACAGGTTCAGGAAG
CPIJ006268	XM_001847617	cardioacceleratory peptide receptor	TCTCGACGCACTTTGTCAGGACTT	ATTGAATCGCTGCTGTGCTGGTTC
CPIJ003873	XM_001845489	beta adrenergic receptor	GCATCGAACGGTTGGCTTCATGTT	ATGATTGCGTACGCCTTGTTGACC
CPIJ014488	XM_001864682	beta adrenergic receptor	TCAACTCGACGCTCAATCCGCTTA	TTTGGAGAAGCAGGGCAGTAGCGAT
CPIJ014409	XM_001864626	alpha-2 adrenergic receptor	GCGGTGGTGAAACCGTTGAAGTTT	GCCGCGTTATTGTTCTTGTTCCGA
CPIJ011118	XM_001861441	allatostatin receptor	CTGCCTTCCACATTGCGTTCTTCT	TTGCTCTTCTTCGACTCTGCGGAA
CPIJ016163	XM_001866351	allatostatin receptor	TCAGCTGAATCCAGACGAGGCAAA	ATGACCTGAATAGGGCACCAGCAA
CPIJ013095	XM_001863362	allatostatin receptor	TTACAACACCACAGCCATCCCAAC	AAGATGGGCACAATCCGTGAAACG
CPIJ017622	XM_001867714	conserved hypothetical protein	TCCCGATGACCCTCATCACCATTT	CAGCCACGACAGCACAAACAGAAT
CPIJ000934	XM_001842552	conserved hypothetical protein	ATCGGCCTATTCATCACGTGGCTA	TCAGCGGGTACAACACCACGATAA
CPIJ006942	XM_00184	conserved hypothetical protein	TGTGACCCAACGGGATCCAAACTA	TCTTGAAGAACACCAGCACCGAGA

	8937			
CPIJ017421	XM_001867456	conserved hypothetical protein	TCTGGTGATTAGCGCGAACCTGAT	TTTCACCGTAAGGCCAGCAGTGTA
CPIJ011756	XM_001861921	conserved hypothetical protein	CAGCAGCAGTTGCAGCTTAAACCA	TGGTTGAGGGTTTCCTGGTCAGAT
CPIJ003683	XM_001845458	5-hydroxytryptamine receptor 2B	AACAACAGCAACCATCAACCACCG	GATTTGGCGTGCTGGCAATGTTCA
CPIJ011755	XM_001861920	conserved hypothetical protein	AGCCATCGATCGCCAAGAAGAAGA	ATCGCGATGATGAAGAACGGCAAC
CPIJ019013	XM_001869365	octopamine receptor oamb	GCCAAAGTTCCAGAGTTCCAAAGT	CTGGAACTCAGAAGTCCTAACTCT
CPIJ019017	XM_001869368	conserved hypothetical protein	TCAGCAGAAGGAGTACTTGACGGT	AAGGGCAACCAACACAGGATGAAC
CPIJ016091	XM_001866585	conserved hypothetical protein	TCCCATGTAATCATCAGCCTGTGC	GCGTGATGATGGTGGCAAAGTACA
CPIJ016092	XM_001866586	adenosine A2 receptor	TCATGATCTGCTGGATTCCGCTGT	ATGCTCATGATCAGATTGCGCAGC
CPIJ019039	XM_001869192	conserved hypothetical protein	TACGTACGGTCTGGAAATGGCAGT	TGAAGCAGCTCCAACAACTCCTGA
CPIJ000647	XM_001842358	conserved hypothetical protein	TCATGGGCGTCTTCCTAGTTTGCT	AGATGATCGGATTGAACGCGGAGT
CPIJ0	XM_	d(1a,b) dopamine	AATGGCGTCAAGTACGAAACGTGC	GCAGCAGTTGGACTGCAGTAGATT

00649	00184<br>2360	receptor

"a" Gene identification number in *Culex quinquefasciatus* genome

"b" Genes were annotated by Vector Database.

"c" The primers were designed according to sequences of each of corresponding genes.

Table S2. Relative expression of GPCR and GPCR-related genes in different mosquito strains of *Cx. quinquefasciatus*

Transcript ID[a]	Larva[b]					Adult[c]				
	S-Lab	HAmCq^{G0}	HAmCq^{G8}	MAmCq^{G0}	MAmCq^{G6}	S-Lab	HAmCq^{G0}	HAmCq^{G8}	MAmCq^{G0}	MAmCq^{G6}
CPIJ013660	1.00	1.54±0.08	1.25±0.44	2.10±0.41	2.49±1.11	5.46±0.51	15.91±1.07	19.81±4.54	19.03±2.79	24.44±5.14
CPIJ015294	1.00	0.74±0.15	0.53±0.05	1.70±0.04	0.29±0.16	1.22±0.12	1.95±0.66	1.88±0.69	1.03±0.49	1.00±0.28
CPIJ019015	1.00	1.17±0.18	1.13±0.69	1.61±0.21	0.85±0.11	2.41±0.21	2.49±0.49	3.57±1.82	3.39±0.77	2.02±0.30
CPIJ018698	1.00	1.47±0.28	1.03±0.06	2.91±0.90	0.68±0.19	2.25±0.30	3.7±0.01	2.62±0.69	3.45±1.79	1.25±0.47
CPIJ001044	1.00	0.70±0.25	0.70±0.21	1.30±0.49	0.60±0.25	1.51±0.20	1.22±0.40	1.41±0.42	1.12±0.15	2.52±1.70
CPIJ008330	1.00	0.93±0.04	0.63±0.18	1.10±0.24	1.08±0.25	4.71±3.39	4.13±0.71	9.35±3.75	7.25±0.07	9.85±3.04
CPIJ011883	1.00	0.65±0.07	0.45±0.21	0.90±0.14	0.35±0.21	3.75±0.49	3.25±0.35	4.90±0.99	3.15±0.21	5.10±0.99
CPIJ003188	1.00	0.80±0.07	0.45±0.21	1.10±0.35	0.95±0.21	3.81±1.77	1.91±1.31	3.00±0.14	3.10±1.21	3.20±1.77
CPIJ016039	1.00	0.57±0.04	0.56±0.30	0.71±0.38	0.34±0.16	0.75±0.30	1.35±0.88	0.99±0.37	1.00±0.01	1.50±0.37
CPIJ015747	1.00	0.46±0.07	0.58±0.26	1.12±0.93	0.23±0.06	0.86±0.32	1.01±0.36	0.90±0.46	0.99±0.54	0.80±0.34
CPIJ014487	1.00	0.70±0.01	0.80±0.05	1.02±0.33	0.67±0.01	0.78±0.03	2.03±0.06	0.59±0.19	3.55±1.63	0.65±0.13
CPIJ013218	1.00	0.98±0.18	0.84±0.15	1.30±0.43	1.63±0.10	12.55±4.88	4.59±2.11	4.94±0.19	6.06±0.65	5.62±0.12
CPIJ003421	1.00	1.80±0.21	0.45±0.07	2.20±1.41	2.35±0.21	3.10±1.27	2.90±0.77	3.20±0.01	4.40±0.85	2.10±1.45
CPIJ014065	1.00	0.60±0.01	0.30±0.14	2.30±1.84	0.60±0.28	2.95±1.34	0.90±0.01	0.90±0.14	3.10±1.27	2.00±0.14
CPIJ005574	1.00	2.40±0.14	2.20±0.90	1.80±0.44	2.20±0.71	1.70±1.00	3.70±1.36	1.75±0.21	3.35±0.21	2.70±0.92
CPIJ016281	1.00	1.53±0.27	1.08±0.27	3.24±0.60	0.94±0.18	2.29±0.83	11.66±0.24	7.52±2.04	6.78±1.89	4.01±0.16
CPIJ013069	1.00	1.05±0.07	0.90±0.14	1.35±0.64	0.75±0.35	1.50±0.57	2.70±0.14	1.90±0.01	1.60±0.57	1.95±0.49
CPIJ007187	1.00	3.00±0.85	1.60±0.21	2.80±0.57	1.50±0.42	5.20±0.78	4.90±0.71	5.21±0.71	4.12±0.49	2.90±0.85
CPIJ006984	1.00	1.90±0.01	0.90±0.23	1.40±1.06	1.72±0.21	7.10±0.01	5.40±0.21	5.50±1.97	5.60±0.92	6.20±2.00
CPIJ018265	1.00	1.35±0.55	1.12±0.91	4.82±1.46	2.04±1.12	1.77±0.12	3.06±0.16	1.62±0.06	9.96±1.00	1.68±0.04
CPIJ014670	1.00	0.85±0.21	1.17±0.24	1.63±0.24	0.40±0.28	1.83±0.25	1.60±0.57	4.35±0.91	3.34±1.04	5.67±2.07
CPIJ012071	1.00	1.00±0.28	1.20±0.28	1.40±0.42	0.95±0.35	0.75±0.07	0.80±0.14	0.60±0.14	0.70±0.14	0.80±0.14
CPIJ018504	1.00	1.17±0.10	1.19±0.06	1.28±0.34	1.54±0.60	2.68±0.73	5.94±1.47	1.91±1.03	1.33±0.49	2.63±0.02
CPIJ010469	1.00	1.24±0.23	0.94±0.05	1.08±0.28	1.55±0.36	0.89±0.45	3.64±1.32	1.91±1.43	2.04±1.18	2.55±0.35
CPIJ011191	1.00	0.70±0.07	0.70±0.07	1.70±0.01	0.90±0.14	7.20±0.07	6.25±1.06	8.80±1.41	11.11±1.41	8.60±0.78
CPIJ003158	1.00	2.05±0.25	0.50±0.20	2.15±0.37	0.90±0.25	9.20±1.39	7.00±1.00	9.30±1.53	11.50±3.54	22.00±6.08
CPIJ011716	1.00	1.45±0.52	0.75±0.16	4.90±0.14	0.45±0.07	17.50±9.19	13.65±2.33	12.67±2.35	23.26±5.30	9.25±1.90
CPIJ016695	1.00	0.80±0.28	0.60±0.01	1.50±2.40	0.65±0.35	0.95±0.07	1.45±0.35	1.00±0.28	1.50±0.70	1.50±0.42
CPIJ006269	1.00	0.95±0.15	0.50±0.01	1.45±0.78	1.45±0.64	1.15±0.44	1.50±0.32	0.91±0.15	0.65±0.21	0.57±0.12
CPIJ007712	1.00	1.70±0.85	0.85±0.07	1.60±0.14	1.25±0.35	3.30±1.77	2.20±0.14	2.70±1.63	1.85±0.07	2.10±0.49

CPIJ011619	1.00	0.90±0.29	1.41±0.28	3.08±1.31	3.00±1.98	2.23±0.24	0.85±0.01	0.73±0.39	1.04±0.51	1.32±0.03
CPIJ001199	1.00	1.15±0.06	0.95±0.06	0.85±0.21	0.53±0.15	0.70±0.28	0.65±0.21	0.80±0.01	0.60±0.14	0.95±0.14
CPIJ005000	1.00	1.50±0.06	1.20±0.25	2.25±0.21	0.83±0.29	126.00±11.31	143.00±24.00	126.00±8.49	106.00±22.63	150.50±4.95
CPIJ009246	1.00	1.83±0.04	0.60±0.07	1.14±0.76	0.50±0.42	9.43±2.02	16.17±2.74	18.48±11.14	11.86±3.03	13.25±0.35
CPIJ013408	1.00	1.35±0.35	0.80±0.42	1.10±0.71	0.70±0.85	120.00±32.53	87.50±0.71	105.50±45.96	158.00±21.21	122.00±10.00
CPIJ004067	1.00	4.50±1.25	2.10±0.42	1.55±0.07	1.10±0.42	1.20±0.14	1.80±1.14	1.10±0.14	1.30±0.26	2.20±0.14
CPIJ012052	1.00	1.55±0.35	1.05±0.07	1.75±0.49	0.45±0.21	961.00±27.01	1224.00±23.76	1026.00±31.21	1022.00±37.77	1138.00±14.38
CPIJ011571	1.00	1.60±0.60	0.70±0.12	2.50±0.57	0.85±0.07	344.00±30.41	264.00±45.86	313.00±24.75	364.00±13.96	330.00±16.61
CPIJ011573	1.00	1.30±0.21	0.30±0.01	0.80±0.01	0.35±0.21	0.01±0.001	0.01±0.004	0.01±0.004	0.01±0.004	0.02±0.001
CPIJ011574	1.00	1.40±0.07	0.55±0.21	1.35±0.78	0.55±0.21	0.25±0.05	0.20±0.01	0.15±0.07	0.08±0.02	0.17±0.03
CPIJ011983	1.00	0.65±0.21	0.65±0.07	1.40±0.71	0.50±0.28	1.45±0.21	1.25±0.07	1.45±0.21	1.85±0.64	1.60±0.28
CPIJ013056	1.00	1.05±0.07	0.65±0.07	0.65±0.07	0.25±0.01	0.35±0.21	0.15±0.07	0.20±0.14	0.15±0.07	0.10±0.01
CPIJ011419	1.00	1.30±0.28	1.15±0.07	3.30±0.01	1.35±0.21	8.40±3.32	12.50±4.80	14.30±0.57	15.50±1.06	9.40±0.57
CPIJ014334	1.00	0.70±0.01	0.25±0.07	0.60±0.14	0.70±0.42	1.00±0.01	0.60±0.10	1.05±0.17	0.95±0.07	2.30±0.29
CPIJ015116	1.00	0.76±0.34	0.30±0.06	0.83±0.21	0.51±0.01	2.89±0.98	1.59±0.34	1.64±0.53	1.82±0.43	1.42±0.32
CPIJ000143	1.00	0.50±0.23	0.40±0.15	0.90±0.42	0.55±0.21	12.00±2.83	2.20±0.64	4.90±0.60	4.30±0.70	5.60±2.33
CPIJ006507	1.00	1.20±0.51	1.00±0.14	0.80±0.57	0.67±0.31	6.80±1.13	4.30±0.81	4.25±0.35	9.10±2.76	6.90±1.23
CPIJ011547	1.00	1.10±0.01	0.80±0.14	1.20±0.01	0.90±0.42	5.50±0.10	6.90±0.01	11.20±3.68	18.60±4.10	11.00±0.92
CPIJ011549	1.00	1.10±0.14	0.95±0.21	3.10±1.27	0.85±0.21	3.30±0.42	2.30±0.49	2.80±0.28	3.90±0.14	1.80±0.10
CPIJ017258	1.00	1.81±0.24	0.43±0.27	5.65±3.05	1.93±0.28	1.94±0.03	2.11±0.45	1.31±0.31	12.76±1.19	1.60±0.52
CPIJ011559	1.00	1.30±0.07	0.80±0.01	3.80±1.06	1.00±0.14	5.40±0.57	6.80±0.57	7.40±0.99	8.30±1.41	5.35±0.14
CPIJ014419	1.00	0.64±0.33	0.72±0.11	0.79±0.25	2.08±0.28	2.85±0.08	2.80±1.56	9.49±0.97	3.90±2.40	5.65±0.35
CPIJ009749	1.00	1.20±0.28	0.40±0.01	2.60±1.48	0.70±0.14	12.40±3.27	15.00±5.14	11.00±1.70	14.00±5.02	12.30±2.12
CPIJ008822	1.00	1.10±0.49	0.60±0.21	1.20±0.85	0.80±0.35	10.00±2.55	4.85±0.25	12.00±2.55	7.15±1.56	6.50±0.99
CPIJ013929	1.00	0.50±0.14	0.25±0.07	0.50±0.01	0.25±0.07	7.40±0.57	2.65±0.07	2.95±1.06	5.40±1.84	4.05±0.78
CPIJ008641	1.00	2.50±1.91	1.30±0.92	2.10±0.49	1.10±0.49	2.90±0.80	2.50±0.10	6.40±1.45	3.20±1.13	4.60±0.99
CPIJ002466	1.00	1.10±0.35	0.85±0.31	1.10±0.35	0.75±0.35	4.20±0.64	3.30±1.15	5.00±1.71	3.30±0.89	2.40±1.10
CPIJ008111	1.00	1.20±0.71	0.75±0.49	1.15±0.21	0.40±0.14	2.40±0.78	1.55±0.71	2.80±0.49	1.70±0.71	2.15±0.07
CPIJ016970	1.00	1.07±0.23	0.55±0.11	1.61±0.18	0.45±0.10	3.96±1.46	2.11±0.23	1.43±0.18	1.44±0.35	1.13±0.30
CPIJ007717	1.00	0.92±0.54	0.76±0.06	0.75±0.21	2.05±0.09	1.95±0.06	5.36±3.33	6.08±2.66	2.15±1.20	3.60±0.25
CPIJ014671	1.00	0.56±0.20	0.33±0.04	0.64±0.06	0.63±0.05	2.35±0.07	1.95±0.85	0.69±0.16	0.79±0.12	1.95±1.06
CPIJ000410	1.00	0.90±0.17	0.95±0.07	1.10±0.42	1.00±0.35	2.25±0.49	1.45±0.35	1.20±0.35	1.20±0.66	1.11±0.29
CPIJ011757	1.00	1.18±0.25	0.55±0.12	1.11±0.28	0.35±0.21	1.41±0.14	4.03±2.02	2.30±0.13	2.81±0.43	1.25±0.21
CPIJ020007	1.00	0.37±0.09	0.23±0.18	0.59±0.18	0.47±0.23	1.45±0.21	0.90±0.67	0.94±0.27	1.39±0.08	1.49±0.04

CPIJ011105	1.00	0.70±0.49	0.60±0.14	1.90±1.27	1.50±0.71	1.00±0.28	0.90±0.10	0.70±0.14	3.40±0.21	2.10±1.20
CPIJ011106	1.00	0.76±0.49	0.30±0.10	0.73±0.24	0.30±0.13	0.30±0.01	0.40±0.01	0.30±0.19	0.50±0.01	0.50±0.35
CPIJ009459	1.00	0.50±0.14	0.30±0.11	0.60±0.01	0.50±0.14	2.70±1.13	2.10±0.57	2.20±1.42	2.10±0.99	2.00±0.28
CPIJ019111	1.00	1.21±0.01	2.81±0.10	1.10±0.01	1.67±0.11	0.68±0.15	0.73±0.23	0.51±0.21	0.88±0.15	0.77±0.16
CPIJ000002	1.00	1.45±0.07	1.45±0.35	1.75±0.07	1.65±0.17	4.50±2.06	2.80±0.28	3.85±1.06	3.80±0.14	4.65±0.07
CPIJ018293	1.00	0.66±0.14	0.32±0.14	0.58±0.06	0.29±0.01	0.95±0.27	1.54±0.95	0.61±0.13	0.68±0.41	0.82±0.01
CPIJ015979	1.00	0.65±0.01	0.57±0.14	0.73±0.18	0.60±0.39	1.27±0.45	1.99±0.01	1.37±0.08	1.78±0.74	1.36±0.96
CPIJ019916	1.00	1.06±0.31	0.67±0.06	1.23±0.08	1.76±0.86	4.99±0.06	2.69±1.99	1.60±0.64	2.19±0.62	1.78±0.45
CPIJ014753	1.00	0.82±0.16	1.07±0.23	2.48±0.33	2.81±0.07	1.30±0.71	3.49±0.22	3.98±1.15	2.71±0.13	1.61±0.11
CPIJ002213	1.00	0.80±0.14	0.45±0.07	2.15±0.28	1.40±0.26	2.70±0.14	7.10±3.96	5.05±0.07	7.80±0.99	3.45±0.64
CPIJ003422	1.00	1.50±0.26	0.73±0.17	1.45±0.35	1.30±0.14	1.20±0.21	0.90±0.23	1.21±0.32	1.70±0.85	1.20±0.55
CPIJ003420	1.00	1.20±0.15	0.55±0.06	1.30±0.20	0.53±0.06	0.60±0.50	1.10±0.01	1.30±0.42	0.80±0.21	0.75±0.07
CPIJ013535	1.00	0.95±0.07	0.75±0.07	3.05±1.56	1.25±0.21	7.45±0.21	3.75±1.48	5.56±3.54	8.00±0.71	7.35±3.75
CPIJ013536	1.00	1.45±0.64	0.70±0.14	3.60±1.84	0.65±0.49	4.00±0.20	1.75±0.15	4.50±1.00	4.65±0.21	5.25±2.05
CPIJ016679	1.00	1.32±0.01	0.53±0.26	1.44±0.74	1.32±0.96	3.67±0.21	2.12±0.21	3.15±1.63	1.77±0.52	1.65±0.51
CPIJ011717	1.00	0.80±0.57	0.50±0.01	1.50±0.85	0.50±0.42	7.05±1.77	5.10±0.99	3.45±0.07	4.60±1.70	3.10±1.70
CPIJ016293	1.00	1.03±0.47	0.33±0.05	0.96±0.64	0.51±0.15	3.44±0.64	3.41±0.47	2.43±1.75	4.11±1.51	3.68±2.59
CPIJ007715	1.00	1.90±0.21	1.25±0.07	1.75±0.49	0.75±0.35	8.60±0.21	5.80±0.57	8.95±1.34	7.70±0.71	8.05±1.48
CPIJ007716	1.00	1.00±0.38	0.75±0.25	1.00±0.54	0.40±0.14	4.30±1.62	2.70±0.64	3.85±0.07	4.15±0.64	4.00±0.12
CPIJ018158	1.00	0.64±0.04	0.55±0.12	0.97±0.02	1.18±0.04	0.44±0.24	0.62±0.18	0.49±0.14	0.80±0.70	0.36±0.16
CPIJ007681	1.00	1.20±0.42	0.60±0.07	2.80±0.49	0.80±0.71	3.40±1.63	1.80±0.28	1.80±0.78	1.90±0.42	1.30±0.42
CPIJ007676	1.00	3.10±0.28	1.25±0.07	4.90±0.71	1.25±0.07	0.95±0.07	2.45±0.07	1.55±0.21	1.60±0.42	1.25±0.07
CPIJ007677	1.00	4.50±1.41	1.10±0.01	5.50±0.15	2.90±0.28	2.00±1.20	4.05±0.07	3.10±0.85	3.30±0.85	3.85±0.14
CPIJ007682	1.00	1.05±0.28	0.65±0.07	3.15±1.27	1.90±0.01	2.50±0.57	1.30±0.14	1.60±0.42	2.00±1.13	2.00±0.99
CPIJ008820	1.00	9.70±1.13	3.45±1.34	2.65±0.92	1.60±0.01	1.40±0.28	12.70±0.99	11.70±5.09	12.90±1.52	8.90±1.23
CPIJ000651	1.00	1.70±0.35	1.25±0.07	1.60±0.50	0.80±0.42	6.90±0.49	7.90±0.14	11.00±1.41	8.00±0.78	5.85±3.61
CPIJ016678	1.00	0.57±0.17	0.85±0.13	0.75±0.12	0.81±0.45	0.96±0.66	2.62±1.08	1.64±0.01	1.92±0.66	5.78±2.16
CPIJ019566	1.00	0.80±0.22	0.82±0.16	0.70±0.23	1.44±0.18	1.95±0.13	1.24±0.96	0.81±0.38	1.28±0.58	1.93±1.42
CPIJ011881	1.00	0.65±0.07	0.45±0.07	1.10±0.57	0.25±0.07	1.30±0.28	1.60±0.42	1.95±0.07	1.95±0.21	2.15±0.07
CPIJ016282	1.00	1.17±0.71	0.93±0.08	0.81±0.18	0.55±0.11	1.91±0.27	3.32±0.55	1.81±0.28	2.17±0.23	1.14±0.63
CPIJ006268	1.00	0.95±0.51	0.80±0.06	2.10±0.78	0.60±0.01	1.65±0.50	1.40±0.06	1.30±0.40	1.25±0.25	1.41±0.20
CPIJ003873	1.00	1.80±0.32	0.70±0.21	3.80±0.35	1.80±0.16	3.40±0.71	3.10±0.99	4.10±1.41	4.40±0.64	5.00±0.01
CPIJ014488	1.00	0.79±0.36	0.96±0.06	1.94±0.66	0.50±0.28	1.88±0.04	2.70±1.70	7.26±3.80	8.66±0.51	3.00±1.70
CPIJ014409	1.00	1.02±0.25	0.90±0.33	2.20±0.29	1.90±0.46	2.61±0.13	5.40±4.10	10.18±0.88	4.10±1.63	9.72±1.29

CPIJ011118	1.00	1.20±0.35	1.55±0.49	3.90±1.41	2.20±0.92	12.30±1.70	12.30±2.76	23.70±1.70	27.50±3.46	11.70±0.42
CPIJ016163	1.00	0.30±0.24	0.52±0.09	0.75±0.20	0.28±0.02	2.22±0.17	2.28±0.08	1.78±0.90	2.24±0.56	3.86±0.16
CPIJ013095	1.00	1.05±0.21	0.95±0.35	1.85±1.34	0.55±0.35	2.50±1.70	3.65±0.49	2.60±1.13	5.10±2.83	2.65±1.06
CPIJ017622	1.00	2.32±0.02	1.58±0.55	1.58±0.17	0.72±0.18	4.36±1.81	3.67±1.27	3.09±1.90	44.25±9.80	2.23±0.07
CPIJ000934	1.00	0.50±0.32	0.80±0.35	0.90±0.26	0.50±0.32	2.10±0.29	3.10±1.30	2.50±1.03	2.90±0.20	7.40±2.33
CPIJ006942	1.00	1.50±0.28	1.40±0.35	2.25±0.71	0.90±0.31	1.15±0.14	1.50±0.21	1.60±0.25	0.95±0.21	1.95±0.35
CPIJ017421	1.00	1.78±0.39	0.46±0.33	1.09±0.31	0.66±0.19	2.95±0.33	3.20±0.91	1.73±0.49	10.32±0.51	1.77±0.0.1
CPIJ011756	1.00	0.44±0.63	0.80±0.28	0.83±0.04	0.30±0.28	0.71±0.27	1.95±0.36	3.30±1.27	0.69±0.27	1.80±0.01
CPIJ003683	1.00	1.70±0.23	0.60±0.23	1.70±0.21	1.15±0.21	2.10±0.85	1.70±0.42	2.60±0.64	3.00±1.27	2.70±0.92
CPIJ011755	1.00	0.85±0.49	0.65±0.21	1.05±0.49	0.40±0.14	0.65±0.21	1.70±0.71	1.40±0.42	1.50±0.71	1.55±0.78
CPIJ019013	1.00	1.18±0.49	0.67±0.25	0.99±0.30	0.51±0.06	1.32±0.62	2.17±0.19	2.56±0.17	2.69±0.38	2.31±0.78
CPIJ019017	1.00	0.75±0.28	0.66±0.30	1.58±0.31	1.46±0.54	2.23±0.67	0.96±0.36	2.86±1.22	3.26±0.16	1.14±0.28
CPIJ016091	1.00	0.69±0.30	0.76±0.04	1.48±0.19	0.55±0.01	2.17±0.16	2.68±0.26	2.43±0.36	3.72±0.81	2.46±1.01
CPIJ016092	1.00	0.99±0.77	0.75±0.13	1.41±0.16	0.64±0.06	5.53±1.85	5.42±1.87	3.25±0.81	5.74±0.24	4.89±1.08
CPIJ019039	1.00	1.56±0.17	1.18±0.14	1.48±0.07	1.21±0.11	3.93±1.25	8.16±2.03	5.52±0.03	2.06±0.01	2.52±0.26
CPIJ000647	1.00	1.10±0.07	0.50±0.21	0.65±0.06	0.65±0.21	2.31±0.56	1.55±0.14	2.15±0.72	1.73±0.32	0.90±0.14
CPIJ000649	1.00	0.83±0.25	0.60±0.14	0.63±0.06	0.75±0.21	3.10±0.14	1.30±0.20	0.75±0.14	1.30±1.34	0.40±0.17

"a" Gene identification number in *Culex quinquefasciatus* genome

"b" 4th instar larva

"c" 3-days old adult without blood feeding

Table S3. Oligonucleotide primers used in dsRNA and qRT-PCR reactions

Transcript ID	Gene	Primer name	Primer sequence
CPIJ019111	conserved hypothetical protein	dsRNA CPIJ019111 F	5’TAATACGACTCACTATAGCGGAGGGCAAGTGCATATAA3’
		dsRNA CPIJ019111 R	5’TAATACGACTCACTATAGTCAAAGACGTGTGGCTACTG3’
CPIJ007717	conserved hypothetical protein	dsRNA CPIJ007717 F	5’TAATACGACTCACTATAGGTATGGCTGGTGGCACTT3’
		dsRNA CPIJ007717 R	5’TAATACGACTCACTATAGCCGTGAAATCCGGTGCT3’
CPIJ014419	calcitonin receptor	dsRNA CPIJ014419 F	5’TAATACGACTCACTATAGCCTCAAATGTGCCCGGATAA3’
		dsRNA CPIJ014419 R	5’TAATACGACTCACTATAGACGAAGAGGCCCTGAAATG3’
CPIJ014334	pteropsin	dsRNA CPIJ014334 F	5’TAATACGACTCACTATAGGCCACCACCTACATCATCTATC3’
		dsRNA CPIJ014334 R	5’TAATACGACTCACTATAGCATCTTCAGCCGGTTCTTCT3’
CPIJ019111	conserved hypothetical protein	RT-RNAi CPIJ019111 F	5’CCATAAACTCCGCCCTTCTT3’
		RT-RNAi CPIJ019111 R	5’GCGTATTCATCTCGTTGGATTTC3’
CPIJ007717	conserved hypothetical protein	RT-RNAi CPIJ007717 F	5’TTCGTTGTATGGCTGGTGGCACTT3’
		RT-RNAi CPIJ007717 R	5’TCATCGGAATGAAGAAGGAGCCCA3’
CPIJ014419	calcitonin receptor	RT-RNAi CPIJ014419 F	5’GAGCGGGTTTACGAAACGATA3’
		RT-RNAi CPIJ014419 R	5’CTTGACTTGTGCGATCACCT3’
CPIJ014334	pteropsin	RT-RNAi CPIJ014334 F	5’GGAAACGCAAACCCTGAAC3’
		RT-RNAi CPIJ014334 R	5’GACAATGATGTTGGTGTAGCTG3’
CPIJ014218	P450CYP9M10	qRTP450-1CxF	5’ATGCAGACCAAGTGCTTCCTGTAC3’
		qRTP450-1CxR	5’AACCCACTCAACGTATCCAGCGAA3’
CPIJ010543	P450CYP9J40	qRTP450-23CxF	5’ACCCGAATCCGGGCAAGTTTGAT3’
		qRTP450-23CxR	5’AACTCCAAACGGTAAATACGCCGC3’
CPIJ010546	P450CYP9J34	qRTP45010546F	5’ATCCGATGTCGGTAAAGTGCAGGT3’
		qRTP45010546R	5’TGTACCTCTGGGTTGATGGCAAGT3’
CPIJ005959	P450CYP6AA7	qRTP4505959F	5’ATGACGCTGATTCCCGAGACTGTT3’
		qRTP4505959R	5’TTCATGGTCAAGGTCTCACCCGAA3’
